# Phosphorylation of CREB at Serine 142 and 143 Is Essential for Visual Cortex Plasticity

**DOI:** 10.1523/ENEURO.0217-21.2021

**Published:** 2021-10-26

**Authors:** Nisha S. Pulimood, Minerva Contreras, Molly E. Pruitt, Agnieszka Tarasiewicz, Alexandre E. Medina

**Affiliations:** 1 Department of Pediatrics, University of Maryland School of Medicine, Baltimore, MD 21201; 2 Department of Physiology, University of Maryland School of Medicine, Baltimore, MD 21201

**Keywords:** ARC, CREB, gene expression, neuronal plasticity, ocular dominance, VEP

## Abstract

The transcription factor cAMP response element-binding protein (CREB) is involved in a myriad of cellular functions in the central nervous system. For instance, the role of CREB via phosphorylation at the amino-acid residue Serine (Ser)133 in expressing plasticity-related genes and activity-dependent neuronal plasticity processes has been extensively demonstrated. However, much less is known about the role of CREB phosphorylation at Ser142 and Ser143. Here, we employed a viral vector containing a dominant negative form of CREB, with serine-to-alanine mutations at residue 142 and 143 to specifically block phosphorylation at both sites. We then transfected this vector into primary neurons *in vitro* or intracortically injected it into mice *in vivo*, to test whether these phosphorylation events were important for activity-dependent plasticity. We demonstrated by immunohistochemistry of cortical neuronal cultures that the expression of Arc, a known plasticity-related gene, requires triple phosphorylation of CREB at Ser133, Ser142, and Ser143. Moreover, we recorded visually-evoked field potentials in awake mice before and after a 7-d period of monocular deprivation (MD) to show that, in addition to CREB phosphorylation at Ser133, ocular dominance plasticity (ODP) in the visual cortex also requires CREB phosphorylation at Ser142/143. Our findings suggest that Ser142/143 phosphorylation is an additional post-translational modification of CREB that triggers the expression of specific target genes and activity-dependent neuronal plasticity processes.

## Significance Statement

The transcription factor cAMP response element-binding protein (CREB) triggers the expression of numerous different gene clusters in response to different cellular stimuli. Previous studies have shown that CREB can be activated by phosphorylation at several of its serine residues. We discovered that ocular dominance plasticity (ODP), a type of activity-dependent plasticity in the visual cortex, requires the phosphorylation of three different serine residues on CREB (Ser133, Ser142, and Ser143). The expression of the critical early gene Arc also requires this triple phosphorylation pattern. Elucidating such phosphorylation patterns of CREB required for activity-dependent gene expression could help us better understand the mechanisms of neuronal plasticity.

## Introduction

cAMP response element-binding protein (CREB) has long been known as a master regulator of neuronal plasticity (Barco and Marie, 2011). It has strongly been implicated in critical cellular phenomena like long-term potentiation (LTP) and other gain-of-function processes like spine expansion and dendritic sprouting (Barco et al., 2002; Middei et al., 2012). Activation of CREB can be attained by different post-translational modifications. Phosphorylation at serine (Ser)133 on CREB’s kinase-inducible domain (KID domain) is critical for activation of CREB and expression of its downstream targets (Mayr and Montminy, 2001). Many different kinases such as CaMKII, CaMKIV, PKA, and MEK can phosphorylate CREB at Ser133 in response to stimuli such as calcium influx, growth factors, mitogen/stress signaling molecules, etc. (Mayr and Montminy, 2001; Johannessen et al., 2004). Therefore, stimulus-specific mechanisms of CREB activation must exist to confer specificity on which genes are expressed to achieve a desired cellular response. In line with this idea, phosphorylation at Ser133 alone is often not sufficient for the expression of many CREB-dependent genes (Kornhauser et al., 2002). For instance, BDNF is regulated by CREB, but its expression does not follow the time course of CREB phosphorylation at Ser133. At early time points after membrane depolarization, CREB is highly phosphorylated at Ser133, but BDNF transcription is not yet induced, and at times when BDNF transcription has shut off again Ser133 phosphorylation is still maintained. This suggests that the phosphorylation of CREB at Ser133 may be important, but not sufficient, for calcium induction of BDNF transcription (Tao et al., 1998). A second piece of evidence for the insufficiency of Ser133 phosphorylation for CREB-dependent gene expression is that different stimuli induce phosphorylation at Ser133 with different kinetics resulting in the expression of different genes (Bonni et al., 1995; Mayr and Montminy, 2001). The growth factors NGF and EGF both trigger phosphorylation at Ser133, but NGF evokes prolonged phosphorylation and expresses the CREB-dependent target VGF, whereas EGF evokes transient phosphorylation and does not lead to expression of VGF. This implies that there needs to be another event in addition to Ser133 phosphorylation to activate transcription of specific pools of CREB-dependent genes (Bonni et al., 1995).

In addition to the best-studied residue Ser133, CREB contains numerous other phosphorylation sites that serve to regulate transcription of its downstream targets (Fiol et al., 1994; Sun and Maurer, 1995; Shanware et al., 2007; Sakamoto et al., 2010). Ser142 and Ser143 (Ser142/143) are two sites on CREB capable of being phosphorylated *in vitro* by CaMKII (Sun et al., 1994) and casein kinase II (Parker et al., 1998) in an activity-dependent manner. These two serines become phosphorylated in response to various stimuli in different regions of the brain, noxious stimuli like formalin in the spinal cord, light and circadian rhythms in the suprachiasmatic nucleus, and calcium influx in cortical neurons (Gau et al., 2002; Kornhauser et al., 2002; Niederberger et al., 2007). While Ser133 is phosphorylated in response to cAMP and depolarization-induced calcium influx, Ser142/143 phosphorylation seems to be insensitive to changes in cAMP signaling (Kornhauser et al., 2002), showing that these CREB activation mechanisms respond to different synaptic stimuli. Preventing phosphorylation at both these sites together impairs CREB-dependent gene expression (Kornhauser et al., 2002). Taken together, these pieces of evidence suggest that Ser142/143 phosphorylation could lend specificity to CREB activation.

### Importance of CREB in ocular dominance plasticity (ODP)

ODP is a paradigm of neuronal plasticity in the primary visual cortex, comprising cortical changes that take place after depriving one eye of patterned visual stimulation (Hubel and Wiesel, 1970). In this paradigm, a monocular deprivation (MD) by eyelid suture is performed, during which cortical neurons responding to the deprived and the experienced eye respectively decrease and increase their responses. ODP therefore encompasses a depression component (Dc-ODP) and a potentiation component (Pc-ODP). In mice, Dc-ODP and Pc-ODP are expressed in a temporally distinct manner, such that Dc-ODP is seen after 3 d of MD, whereas Pc-ODP only appears after at least 5–7 d of MD (Frenkel and Bear, 2004).

In the visual cortex of the ferret, blocking phosphorylation of CREB at Ser133 blocked the ocular dominance shift in the ferret visual cortex after 3 d of MD, as measured by single-unit recordings *in vivo* (Mower et al., 2002). Recently, we extended this finding to mice, using visually-evoked potential (VEP) recordings in awake animals before and after 7 d of MD, and showing that CREB is required for both Pc-ODP and Dc-ODP *in vivo* (Pulimood et al., 2017). In light of the role of Ser142/143 phosphorylation in activity-dependent expression of CREB target genes, we hypothesized that the phosphorylation of CREB at Ser133 is not sufficient for ODP, and additional phosphorylation at Ser142/143 is required.

## Materials and Methods

### Animals

Wild-type C57BL/6 mice between the ages of postnatal day (P)25 and P35 were used for the *in vivo* electrophysiology to restrict recordings to the critical period of visual cortex development. Sprague Dawley rat pups at embryonic day (E)20 were used to make cortical cultures for the *in vitro* experiments. All animals were used in accordance with the protocols of the University of Maryland School of Medicine Institutional Animal Care and Use Committee.

### Herpes simplex virus (HSV) constructs

Two different CREB dominant-negative (CREBdn) plasmid constructs were packaged in HSV vectors. These constructs contain a GFP tag and have serine to alanine point mutations at the residue 142 and 143 (CREBdn-S142A/S143A) or 133 (CREBdn-S133A), which prevents phosphorylation of CREB at these sites. The control viral construct (HSV-GFP) expressed GFP alone. All the HSV constructs were generated by Rachel Neve at the Massachusetts Institute of Technology Viral Core. Virus titer of GFP and CREBdn-S133A was 6 × 10^7^ transducing units/ml, and titer of CREBdn-S142A/S143A was 3 × 10^8^ transducing units/ml.

### Virus infection and KCl stimulation of cortical cultures

Cortical cultures were prepared in the lab of our collaborator, Thomas Blanpied, at the University of Maryland School of Medicine. Briefly, primary cultures of cortical neurons were obtained from E20 rat embryos and dissociated with trypsin. On the day of dissection, the cells were grown in Neurobasal Medium (Sigma) supplemented with 5% bovine serum supplemented with B27, glutamax and 50 U/ml gentamicin. Cells were plated at 50,000 cells/cm^2^ on coverslips coated with poly-L-lysine (Sigma). The next day, the media was changed to Neurobasal medium (Sigma) supplemented with B27, glutamax, and 1 μg/ml gentamycin. FUDR (10 μm) was added 1–3 d after plating. Cultures were grown at 37°C and in 5% CO_2_.

After 10 d in culture, a 1:2000 dilution of HSV construct (HSV-GFP, CREBdn-S133A, or CREBdn-S142A/S143A) was added to each well. Plates were returned to the 37°C incubator overnight, and virus infection was confirmed the following day by visualizing GFP expression in the culture plate. Cells were stimulated for 20 min either in a potassium chloride (KCl)-rich media containing artificial CSF (ACSF) with 50 mm KCl and 9.2 mm CaCl_2_ as previously described (Kornhauser et al., 2002) or normal ACSF (Ma et al., 2014) as the control. The KCl-rich media additionally contained 0.5 μm tetrodotoxin (TTX; to block voltage-gated sodium channels), 10 μm DNQX (to block AMPA receptors), and 10 μm APV (to block NMDA receptors; Ma et al., 2014). These blockers decreased the baseline activity level, so that any activity-dependent change in expression of our proteins of interest could be clearly resolved.

KCl-stimulated cells used to stain for phospho-CREB (pCREB) were fixed immediately after stimulation with 4% paraformaldehyde (PFA) in 20% EGTA. However, stimulated cells used to stain for CREB target genes were switched back into culture media for 20 min to allow for gene expression, before subsequent fixation.

### Western blotting

The HSV construct was intracortically administered into Layer IV of the visual cortex of mice via stereotaxic injection (see below, Electrode implantation and intracortical injection). The animals were killed after 2–4 d (time allowed for viral infection) with isoflurane followed by decapitation. The visual cortex was dissected out on ice and GFP fluorescence was confirmed by microscopy. The GFP-positive tissue was isolated and homogenized in RIPA lysis buffer (Millipore 20-188) with protease/phosphatase inhibitors (Cell Signaling 5872). Protein concentrations were determined by Bradford assay and samples were run on 15% TGX Protean gels (Bio-Rad), in the mini-protean Bio-Rad Tetracell electrophoresis chamber. Gels were transferred to PVDF membranes using the Bio-Rad Trans-blot turbo transfer system. Membranes were blocked for at least 90 min using 2–4% non-fat blotting-grade blocker (Bio-Rad) in 1× Tris-buffered saline with 0.1% Tween (1× TBST), then incubated overnight at 4°C with a 1:1000 dilution of rabbit anti-pCREB-133 (pCREB-133; Millipore catalog #06-519). After washing three times in 1× TBST, membranes were incubated for 1 h in horseradish peroxidase-conjugated anti-rabbit IgG at (Cell Signaling Technology catalog #7074) at a 1:3000 dilution. ECL reagents (Bio-Rad CLARITY) were used to chemiluminescently visualize the protein on an imaging system (ProteinSimple FluorChem HD2). ImageJ (RRID: SCR_003070) was used for densitometry and all OD values were normalized by loading control. Cyclophilin B (Thermo Fisher Scientific catalog #PA1-027A) was used as the loading control in all cases. As soon as the imaging of pCREB-133 was complete, the membranes were washed in stripping buffer for 15 min at room temperature and blotted again for total CREB (1:5000 dilution of rabbit anti-CREB; Millipore catalog #04-218) using the same blotting procedure described above. Arc expression was assessed by similar procedures, antibody used was catalog #66550-1-ig from proteintech at 1:5000 concentration.

### Immunocytochemistry

Fixed cells were washed with 1× PBS and 100 mm glycine (PBS/Gly), and then blocked in 10% normal goat serum (NGS) in PBS/Gly with 0.1% Triton X-100 for 60 min at 37°C. They were then incubated at 4°C overnight with the following primary antibodies in PBS/Gly/0.1% Triton X-100 in 5% NGS: rabbit anti-pCREB Ser133 (1:1000, Millipore), rabbit anti-pCREB Ser142/143 (1:500), rabbit anti-Arc (1:500, Santa Cruz), rabbit anti-CaMKII conjugated to Alexa Fluor 647 (1:500, Abcam). Each coverslip of cells was stained for either pCREB Ser133, pCREB Ser142/143, or Arc, together with CaMKII as an excitatory neuronal marker. The next day, the cells were washed in PBS/Gly, incubated for 1 h at room temperature with fluorescent secondary antibody anti-rabbit Alexa Fluor 568 (1:500), washed again in PBS/Gly, and then mounted on glass slides with mounting media (Permafluor mountant, Thermo Scientific). The pCREB Ser142/143 antibody was generously provided to us by Michael Greenberg of Harvard University.

### Confocal imaging and analysis

Confocal imaging was performed at the University of Maryland School of Medicine Confocal Microscopy Core Facility, on a point-scanning confocal (Zeiss LSM 510 Meta) microscope, with a 40×/1.3 NA oil-immersion objective. Specifications for data collection on each fluorescent track were as follows: for GFP, 488-nm laser excitation was bandpassed from 500 to 550 nm; for Alexa Fluor 594, laser excitation at 543 nm was set to a long-pass filter at 560 nm; and for DAPI, 730-nm pulsed two-photon laser excitation was bandpassed at 380–550 nm. Colocalization analysis was performed on maximum projection images. Using the Grid and Cell Counter tools in ImageJ software, all GFP-positive cells (GFP = virus expression) that co-expressed CamKII (Alexa Fluor 594; CamKII = marker of excitatory neurons) were manually selected and counted. Only virus-infected, excitatory neurons with clear somatic boundaries and a visible nucleus were included in this analysis, determined by colocalization of GFP and CaMKII. The localization of CamKII in the cytosol and dendrites allowed us to clearly delineate the nucleus of every cell analyzed. To determine nuclear staining, we used ImageJ to specifically define the nuclear region as the region of interest (ROI), and then quantified the CREB fluorescence within each ROI. The average optical density (OD) of the ROI was determined, and the background fluorescence was subtracted from the OD value of each cell.

### Electrode implantation and intracortical injection

Surgery was conducted on mice under isoflurane anesthesia. Burr holes were drilled in the skull at 0.5 mm rostral to λ and 3 mm lateral to the midline, which corresponds to the binocular zone of the visual cortex (V1B) in mice. A microsyringe pump-controller (World Precision Instruments Micro4) was used to bilaterally deliver 1 μl of the desired HSV construct into Layer IV of the cortex at an infusion rate of 1 nl/s, with a diffusion time of 2–3 min. Tungsten electrodes (FHC) with tip impendences between 0.3 and 0.55 MΩ were stereotaxically implanted bilaterally in the same location as the injection, at a depth of 450–480 μm, to target Layer IV cells as previously described (Porciatti et al., 1999; Heynen and Bear, 2001; Lantz et al., 2015; Pulimood et al.,2017). Reference electrodes were implanted at ∼0.5 mm caudal to bregma and 2 mm lateral to the midline. The four electrodes as well as a vertical post (for immobilization during VEP recordings) were secured to the skull with cyanoacrylate, creating a fixed headstage from which chronic VEP recordings were made.

### Monocular Deprivation

Surgery was conducted under isoflurane anesthesia, following the baseline VEP recording. Ophthalmic proparacaine (Akorn, Inc) was applied topically, and the edges of the upper and lower eyelids trimmed. The lids were stitched together using 7–0 prolene suture (Ethicon, Inc), and Gluture tissue glue (Abbott Laboratories) sealed the lids together. A thin film of cyanoacrylate (tissue glue) was used to cover the area to protect the surgical site during the 7-d period of MD.

### VEP recordings

This VEP recording procedure was performed the same way for both pre-MD and post-MD recording sessions. The animals’ heads were immobilized so that movement artifact was kept to a minimum during electrophysiological recordings. Mice were then presented with a visual stimulus to each eye independently, during which visually-evoked local field potentials were recorded. Recordings were conducted using XCell-3 amplifiers (FHC), a 1401 digitizer (Cambridge Electronics Design), and Spike 2 software (Cambridge Electronics Design). XCell-3 amplifiers were set at a low-frequency cutoff of 0.1–10 Hz and a high-frequency cutoff of 100 Hz. The visual stimulus consisted of a full-field, phase-reversing, ordinal sine grating at 0.5 Hz with 100% contrast. The stimulus was controlled by a custom program written in MATLAB (The MathWorks) and presented at a distance of 21 cm from the animal. The angle of the stimulus grating was changed (45° to 135°) for sessions before and after MD, to avoid any confounding results with respect to stimulus-selective response potentiation (Cooke and Bear, 2010). Recordings were made of at least 100 stimulus presentations, and peak to trough amplitudes were measured. As in previous studies, compensation for variations in noise and impedance was conducted (You et al., 2011; Makowiecki et al., 2015). The average distance of each data point from the mean of all data points in each analyzed recording was determined for both pre-MD and post-MD recordings. A ratio of this mean difference was then multiplied by the peak-to-trough measure of the post-MD field potential to calculate the post-MD VEP amplitude. The contralateral bias index (CBI) was calculated as the ratio of the contralateral VEP amplitude over the ipsilateral VEP amplitude for each animal.

### Statistics

VEP experiments assessing ODP (CBI or VEP amplitudes) were analyzed using directional paired *t* tests because this *in vivo* technique allows for within-subject controls. One-way ANOVA was used to analyze differences in VEP amplitudes and CBI between naive mice and each virus-infected group. For immunocytochemistry, pCREB was compared using two-tail Student’s *t* tests, whereas Arc and CaMKII were compared using one-way ANOVA. Western blottings were analyzed by two-tail Student’s *t* tests. All statistical tests were performed on IBM SPSS (v23) and statistical significance was set to *p* ≤ 0.05, or *p* < 0.03 (with Bonferroni correction), and denoted by an asterisk (*). For clarity, statistical details are reported in the figure legends.

### Disclosure

All experiments were conducted at the same time and by the same investigator. The GFP-control animals used in the VEP experiments and for Western blot tissue were the same as those reported in our previous study published in *J Neurosci* (Pulimood et al., 2017). As required by *J Neurosci*, any of these previously published data are explicitly labeled as such, with the corresponding figures enclosed in gray boxes.

## Results

In this study, we tested the hypothesis that CREB phosphorylation at Ser142/143 is required for at least one, if not both components of ODP, Dc-ODP and Pc-ODP. We used viral-mediated genetic blockades and *in vivo* electrophysiology, predicting that a blockade of CREB phosphorylation at Ser142/143 will disrupt ODP *in vivo.*

### Neuronal activity leads to phosphorylation of CREB at Ser142/143

**Figure 1.**
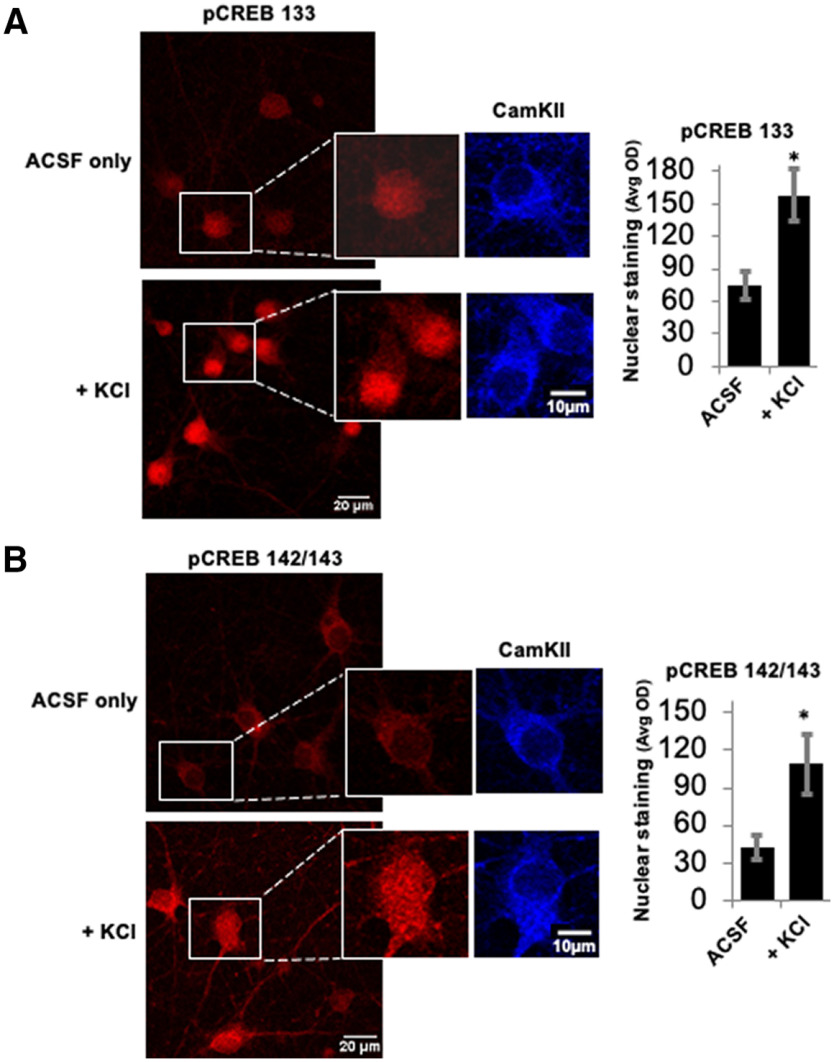
Phosphorylation of CREB at Ser133 and Ser142/143 is activity dependent. ***A***, Cells exposed to either regular ACSF or KCl-rich ACSF for 20 min show an increase in staining intensity of pCREB 133 (red). The inset shows a higher-magnification image where nuclear staining of pCREB133 is increased after depolarization with KCl. CaMKII staining (blue) is not in the nucleus under either stimulus condition. The histogram on the right displays the average optical density (Avg OD), quantifying the change in nuclear intensity of pCREB133 (*n* = 3 experiments, 12 coverslips, 137 cells for the ACSF group and 3 experiments, 13 coverslips, 141 cells for the +KCl group; *p < 0.0001, independent *t* test). ***B***, Cells stained for pCREB 142/143 (red) also show KCl-induced increase in nuclear staining, whereas CaMKII (blue) in the same cells does not. The inset shows a higher-magnification image and the corresponding histogram represents pCREB142/143 nuclear staining (*n* = 3 experiments, 15 coverslips, 176 cells for the ACSF group and 3 experiments, 9 coverslips, 87 cells for the +KCl group; *p < 0.0001, independent *t* test). All error bars indicate standard error of the mean (SEM).

A primary requirement for any potential mechanism of ODP is that it is activity dependent. Phosphorylation of CREB at Ser133 increases in response to increased synaptic activity (Sheng et al., 1990; Ma et al., 2014). Kornhauser and colleagues showed that phosphorylation at Ser142/143 also increases on synaptic activity (Kornhauser et al., 2002). Therefore, we first aimed to confirm these findings by triggering membrane depolarization with 50 mm KCl for 20 min in dissociated rat cortical cultures, and staining for antibodies that recognize CREB phosphorylation either at Ser133 (pCREB133) or at Ser142/143 (pCREB142/143). We observed that phosphorylation at Ser133 and Ser142/143 (nuclear staining) was increased in cells exposed to KCl-rich ACSF compared with cells exposed to control ACSF (Fig. 1*A*,*B*).

### Expression of Arc requires phosphorylation of CREB at Ser142/143

**Figure 2.**
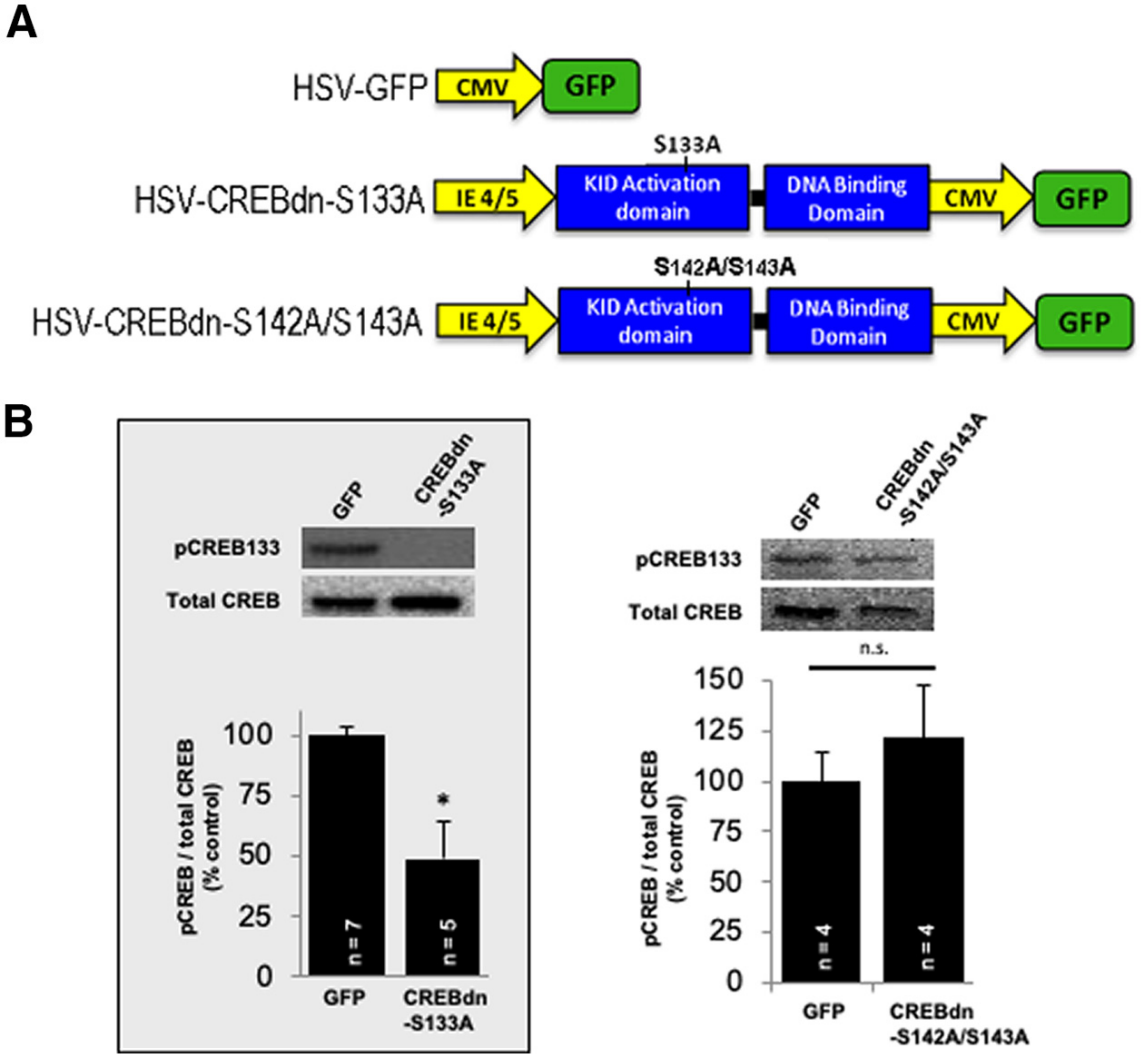
Viral constructs and functional validation. ***A***, Diagrams of HSV constructs used in this study, as described in Materials and Methods. The different domains of the CREB protein are shown in blue, with the dominant negative mutation indicated. The fluorescent tag (GFP) is in the green box. The yellow arrows are the promoters for each gene (IE 4/5 for CREB, and CMV for GFP). ***B***, Western blots showing that phosphorylation at Ser133 is not affected by S142A/S143A mutations. Left, pCREB133 is significantly reduced in tissue from CREBdn-S133A-injected mice versus mice injected with the control virus (*p* = 0.02, independent *t* test). Right, pCREB133 levels do not change in tissue from CREBdn-S142A/S143A-injected mice versus mice injected with the control virus (n.s. = not significant, *p* = 0.49, independent *t* test). The data in the gray box were previously published in *J Neurosci* (Pulimood et al., 2017). All error bars indicate standard error of the mean (SEM).

The fact that phosphorylation of CREB at Ser142/143 is activity-dependent makes it a viable candidate to confer specificity on the activation of CREB and its subsequent expression of diverse gene programs. Arc is an activity-dependent, critical, immediate early gene required for ODP (McCurry et al., 2010; Cohen et al., 2016). Therefore, if Arc expression requires phosphorylation at Ser142/143, it would be likely that CREB phosphorylation at these residues will also be required for ODP. We genetically blocked CREB phosphorylation at Ser142/143 using the HSV construct CREBdn-S142A/S143A **(**Fig. 2*A*). Since Ser133 phosphorylation of CREB is known to be required for Arc expression (Kim et al., 2013; Chen et al., 2017), we conducted Western blottings to confirm that the effects of CREBdn-S142A/S143A on Arc expression did not originate from interference with phosphorylation at Ser133 (Fig. 2*B*; Pulimood et al., 2017).

**Figure 3.**
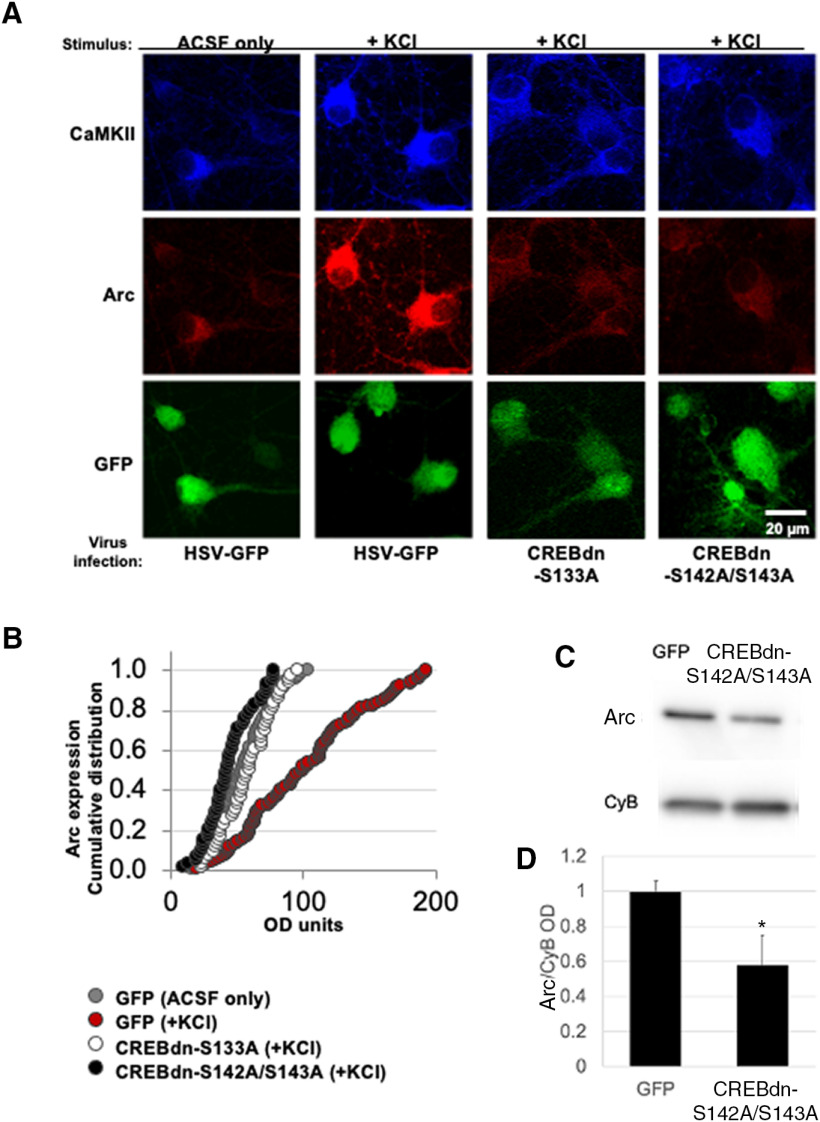
Activity-dependent Arc expression is blocked in the absence of CREB phosphorylation at Ser133 as well as at Ser142/143. ***A***, Stimulus and virus infection conditions are as follows (from left): ACSF control media in cells infected with HSV-GFP control virus (*n* = 105 cells on 12 coverslips from 3 independent experiments), KCl-rich ACSF to depolarize cells infected with HSV-GFP control virus (*n* = 128 cells on 15 coverslips from 3 independent experiments), KCl-rich ACSF in cells infected with CREBdn-S133A (*n* = 51 cells on 20 coverslips from 3 independent experiments), and KCl-rich ACSF in cells infected with CREBdn-S142A/S143A (*n* = 63 cells on 15 coverslips from 3 independent experiments). Top panels show Arc staining in red and bottom panels show HSV infection in green. ***B***, A cumulative distribution plot showing all analyzed cells in each condition clearly displays the activity-dependent increase in Arc expression that is blocked by the CREBdn viruses [one-way ANOVA, *F*_(3,333)_ = 53.1, *p* < 0.0001; Tukey’s *post hoc* test GFP(+KCl) vs all other groups *p* < 0.0001]. ***C***, Representative case of Arc expression in mice injected with CREBdn-S142A/S143A or control GFP. Cyclophilin B (CyB) was used as a loading control. ***D***, Quantification shows a significant reduction in Arc expression after blocked phosphorylation of CREB at Ser142/143 (*t* = 2.32, *p = 0.04; df = 8, *t* test for unequal variances). All error bars indicate standard error of the mean (SEM). OD = optical density.

We infected cells in culture with a control virus (HSV-GFP) or a virus that blocked phosphorylation at Ser133 (CREBdn-S133A) or phosphorylation at Ser142/143 (CREBdn-S142A/S143A). After confirming successful virus expression by GFP visualization, we depolarized these cells with KCl to measure the change in activity-dependent Arc expression. Since mechanistic differences do exist with respect to cell type, we refined our experimental design by co-labeling these cells with CaMKII. As CaMKII is expressed only in glutamatergic neurons and not inhibitory neurons in the cortex (Liu and Jones, 1996), the colocalization of GFP and CamKII allowed us to visualize Arc expression only in virus-infected, excitatory neurons. While neurons infected with HSV-GFP control virus showed a large increase in Arc expression after KCl-induced depolarization, the ones infected with CREBdn-S133A or CREBdn-142/143 did not (Fig. 3*A*). This result demonstrated that a triple phosphorylation of CREB is needed for Arc expression. A cumulative frequency distribution quantifying the above results showed a significant increase in Arc expression only when KCl was applied in cells expressing GFP but not in cells expressing CREBdn-S133A or CREBdn-142/143 (Fig. 3*B*). Taking these results together, our findings in culture strengthened our prediction that phosphorylation at Ser142/143 is a critical secondary event (in addition to phosphorylation at Ser133) for the expression of CREB-dependent genes required for ODP *in vivo*.

To confirm our findings observed in the aforementioned experiments in culture we also tested whether blocking CREB phosphorylation at Ser142/143 would reduce Arc expression *in vivo*. Mice received intracortical injections of CREBdn-S142A/S143A (*n* = 7) or control GFP (*n* = 9) and were monocularly deprived for 3 d to induce Arc expression (Tagawa et al., 2005) and tissue collected. Figure 3*C*,*D* shows a significant reduction of Arc expression in mice receiving CREBdn-S142A/S143A when compared with controls (*t* = 2.32, *p* = 0.048). In summary, blockade of CREB phosphorylation at Ser142/143 reduced Arc expression both *in vitro* and *in vivo*.

### Phosphorylation of CREB at Ser142/143 is required for ODP

**Figure 4.**
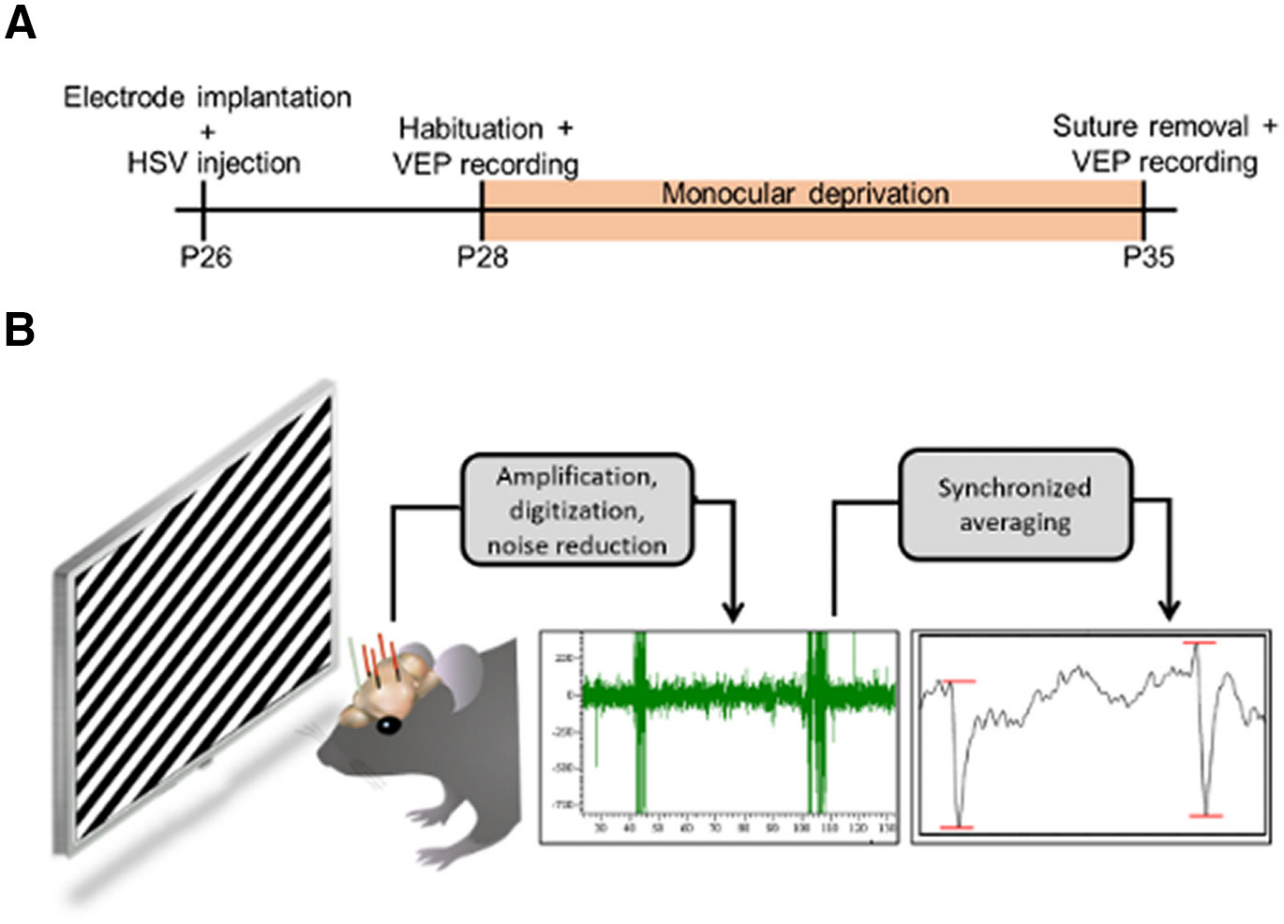
VEP recordings: schematics of the experimental timeline and spike analysis. ***A***, Electrode implantation and virus injection were done together at P26. After recovery from surgery, habituation followed by a pre-MD baseline recording was performed. The animal was then monocularly deprived by eyelid suture for a 7-d period, after which the deprived eye was reopened and a post-MD recording was conducted. VEP = visually evoked potential; HSV = herpes simplex virus; P = post-natal day. ***B***, As the implanted mouse views the visual stimulus, electrical signals are transmitted through an amplifier, noise eliminator, and digitizer, and are recorded as the EEG signal shown in green. The amplitude of the VEP response (after synchronized averaging) is measured in microvolts (μV) from peak to trough as marked by the red lines.

We tested whether ODP is affected by blocking CREB phosphorylation at Ser142/143 using *in vivo* VEP recordings in virus-injected mice before and after 7 d of MD during the critical period of visual cortex plasticity. The chronic implantation of the recording electrodes allowed for a within-subject, before-and-after comparison of evoked field potential amplitudes in the visual cortex. Figure 4 shows a schematic representation of VEP spike acquisition and the VEPs experimental timeline.

This method was similar to what we used in a recent study, where we showed that virus injections targeted to cortical Layer IV resulted in robust infection of cells in Layers IV and II/III, and few cells in deeper Layers V and VI (Pulimood et al., 2017). GFP-marked viral expression was clearly visible within 24 h of injection and could be seen up to 10 d later (Pulimood et al., 2017). We also showed that HSV constructs are neurotropic, infecting primarily excitatory neurons (Pulimood et al., 2017). Therefore, the recording electrodes implanted in Layer IV were able to acquire VEPs from a population of excitatory neurons with viral-mediated blockade of pCREB-Ser142/143.

**Figure 5.**
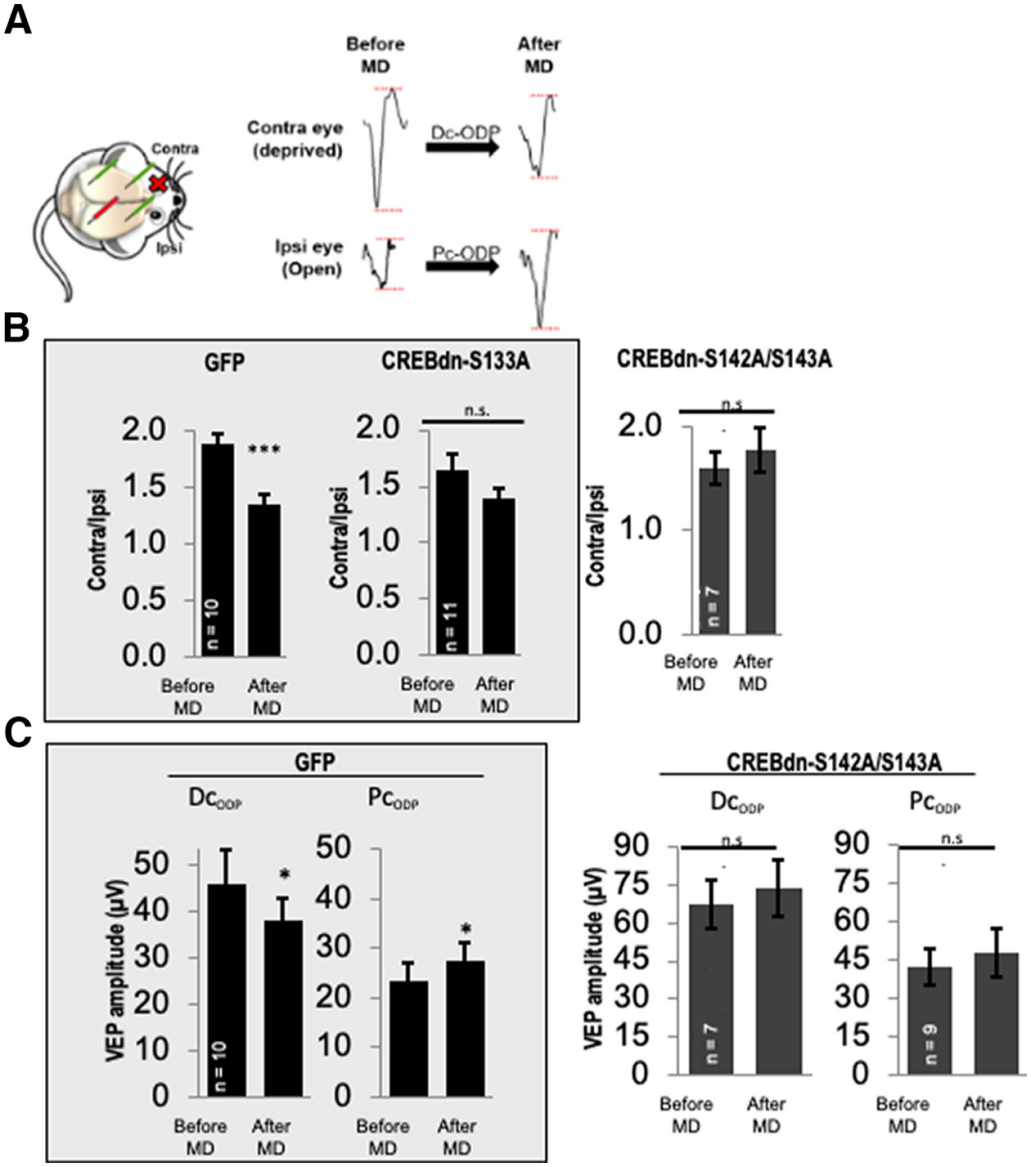
Blocking CREB phosphorylation at Ser142/143 blocks both components of ODP. ***A***, left, An implanted mouse with two recording electrodes and two reference electrodes. The red cross on the mouse’s eye represents the MD, and VEPs are recorded from the hemisphere contralateral to the MD (contra eye deprived; red recording electrode) during stimulation of each eye individually. Right, Representative VEP traces before and after MD, with red lines indicating peak to trough amplitude of the VEP from the deprived eye (sutured closed during MD period) and open eye (remained open during MD period). Comparing responses before and after MD, Dc-ODP represents a decrease in VEP amplitude from stimulation of the deprived-eye stimulation, and Pc-ODP represents an increase in VEP amplitude from open-eye stimulation. ***A***, left, Histogram showing that mice injected with HSV-GFP show the expected downward shift in OD after MD (*p* = 0.0004), whereas mice injected with CREBdn-S133A do not (n.s. = not significant, *p* = 0.07). Right, Mice injected with CREBdn-S142A/S143A also do not exhibit any OD shift (*p* = 0.40). ***C***, left, Histogram showing Dc-ODP and Pc-ODP as expected in control mice (*p* = 0.04 and *p* = 0.01, respectively). Right, Both Dc-ODP and Pc-ODP are blocked in mice injected with CREBdn-S142A/S143A (*p* = 0.45 and *p* = 0.28, respectively). The data in the gray boxes were previously published in *J Neurosci* (Pulimood et al., 2017). Sample sizes (*n*) are specified in the histogram for each group. Data in ***B***, ***C*** were statistically analyzed using paired *t* tests. All error bars indicate standard error of the mean (SEM). Contra = contralateral; Ipsi = ipsilateral; MD = monocular deprivation; Dc-ODP = depression component of ocular dominance plasticity; Pc-ODP = potentiation component of ocular dominance plasticity.

HSV-GFP control animals copied the expected pattern in naive mice (Fig. 5*A*,*B*; Pulimood et al., 2017), with a decrease in VEP amplitude on contralateral eye stimulation (contra) and an increase in the amplitude on ipsilateral eye stimulation (ipsi), whereas CREBdn-S142A/S143A animals showed no change in contra or ipsi VEP amplitude after 7 d of MD (Fig. 5*B*). The CBI, which is the ratio of the VEP amplitude during contralateral eye stimulation over the amplitude during ipsilateral eye stimulation (contra/ipsi), was used as the metric to measure the ocular dominance shift. The inherent contralateral bias of VEPs in mice with normal plasticity in V1B (since most mouse retino-thalamic fibers decussate at the optic chiasm before arriving in the visual cortex) was detected as a downward shift in CBI. While GFP-control mice exhibited an expected decrease in CBI after MD, CREBdn-S142A/S143A did not (Fig. 5*C*). These results were similar to what was observed when blocking phosphorylation of CREB at Ser133 (Pulimood et al., 2017).

Taken together, the novel results presented here reveal the phosphorylation of CREB at Ser142/143 as a novel mechanism of ODP during the critical period, specifically necessary for both components of activity-dependent neuronal plasticity in the visual cortex.

## Discussion

In this study, we confirmed previous evidence (Kornhauser et al., 2002) showing that neuronal activity triggers phosphorylation of CREB at Ser142/143 (Fig. 1). This phosphorylation is required for the expression of critical plasticity-related genes like Arc (Fig. 3) and for ODP (Fig. 5).

Although other studies have shown that CREB induces Arc expression (Kawashima et al., 2009; Sano et al., 2014; Yiu et al., 2011), the mechanism of CREB-dependent Arc expression remained unclear. Arc expression was shown to be decreased in the presence of a dominant-negative form of CaMKII (Kumar et al., 2012). Since CaMKII can phosphorylate CREB at both Ser133 and Ser142/143 (Kornhauser et al., 2002), it is reasonable that CREB-dependent Arc expression may occur via phosphorylation at these sites. We presented direct evidence that triple phosphorylation at Ser133, Ser142, and Ser143 is a mechanism for the activity-dependent induction of Arc by CREB.

It was previously reported that phosphorylation at Ser142 alone can be inhibitory and attenuated CREB-dependent transcription (Sun et al., 1994; Parker et al.,1998). Kornhauser and colleagues expanded this finding to show that Ser142 phosphorylation is inhibitory to CREB-dependent transcription only when it affects CREB dimerization and DNA binding. Using a construct that could not heterodimerize to drive CREB-transcription, they showed that Ser142 phosphorylation in fact enhanced CREB-dependent gene expression (Kornhauser et al., 2002). As opposed to phosphorylation at Ser142 alone, which can be inhibitory under some conditions, phosphorylation at Ser143 alone always led to an increase in CREB-dependent gene expression (Kornhauser et al., 2002). Interestingly, an antibody specific for phosphorylated Ser143 could not detect phosphorylation at this site without phosphorylation at Ser142 (Kornhauser et al., 2002), suggesting that they work in concert. Taken together, these data corroborate our finding that phosphorylation of both Ser142 and Ser143 together induce expression of CREB-dependent genes.

Since the CREB-dependent transcriptome is vast in number and diverse in purpose, the search for which genes CREB expresses to fulfill different cellular needs has been a long-standing question. We showed that triple phosphorylation at Ser133, Ser142, and Ser143 is required for both components of ODP, Dc-ODP and Pc-ODP. Future studies could use transcriptome profiling with these phosphorylation sites blocked, to identify CREB-dependent genes required for both components of activity-dependent plasticity. It would also be important to test whether individually blocking phosphorylation at Ser142 or Ser143 might differentially affect Dc-ODP and Pc-ODP.

## Synthesis

Reviewing Editor: Francisca Bronfman, Universidad Andrés Bello Decisions are customarily a result of the Reviewing Editor and the peer reviewers coming together and discussing their recommendations until a consensus is reached. When revisions are invited, a fact-based synthesis statement explaining their decision and outlining what is needed to prepare a revision will be listed below. The following reviewer(s) agreed to reveal their identity: NONE.

In this paper, the authors provide novel information about the role of the phosphorylation of CREB in Ser 142 and 143 in the expression of Arc, a known plasticity-related gene. In addition, they studied the role of these CREB phosphorylation sites in the visual cortex using the ocular dominance plasticity model. It provides advances in the field and is particularly useful to understand the molecular basis of activity-dependent plasticity in the visual cortex.

Some aspects of the manuscript require modification in the text and figures for re-consider this article for publication.

1) The abstract stays short describing the study without explaining the tools used, such as genetic blockade using a virus as well as the in vivo electrophysiology, etc. There is more space to be more informative. Similar comment for the discussion, more effort should be made to place in context the new findings.

2) There are two more supplementary figures explaining the virus constructs and validation. It would be helpful to add those figures to the main text to guide the reader through the experimental procedures and logical thinking about results. Supplementary Figure 1 could be Figure 2A, for example.

3) Figure legends of supplementary data indicate three figures, but only two figures are found.

4) Section B in supplementary figure 2 is not explained in the figure legend.

5) In the methods describing Western, how GFP of the virus reached the area of interest (that used to guide during dissection)? Was this under the control of a specific promoter? This should be clearly described in the virus description section.

6) Methods section lacks a clear description of cell counting/or OD such as; numbers/experiment for ICC analysis, criteria of choosing cells for analysis, etc. In this manuscript, just the number of cells is described in the figure legends (which is insufficient for such analysis and should be stated in the methods). The number of times the experiments were replicated should also be included- Figure legend should describe n of experiment/groups, not cells. Also, it is not indicated how the nucleus was defined to determine nuclear-increased phosphorylation of CREB (Figure 1). It would be advisable to show nuclear staining or tell how the nuclear area was defined.

7) In figure1, stimulation with potassium triggers Ca2+ influx and therefore activates CAMKII. This should be reflected as a change in CAMKII between stimulated vs control in immunocytochemistry (ICC) of figure 1.

8) Further analysis is needed, at least Western blot to confirm findings of figure 2 about ARC link to CREB. In addition, the early conclusions in the discussion, such as the following statement, need further confirmation. “We presented direct evidence that triple phosphorylation at Ser133, 142, and 143 is a mechanism for the activity-dependent induction of Arc by CREB”.

Minor concerns:

1. Why were cells infected at DIV 10 not at an earlier time point?

2. Typo on page 3 second paragraph “Taken together, these data show that Ser142/143 phosphorylation could lend specificity to CREB activation.”

3. Please revise citations and references; there are typos there too.

4. Please revise the work art of graphs, particularly fig.2, where the line is in the middle of the letter defining axis X, and Figure 3, where the n=of experiments float around and are not in the graph bars. It will be advisable that the format of all the graphs of the figures are the same.

## Author Response

First, we want to thank the Editor and the reviewers for handling our manuscript and for the suggestions. We are pleased that our study was considered to an “an advance to the field” and that has been “judged potentially suitable for publication”. Please see below our response to the reviewer’s comments.

1) The abstract stays short describing the study without explaining the tools used, such as genetic blockade using a virus as well as the in vivo electrophysiology, etc.

We agree with the reviewers and modified the abstract accordingly (bold used for inserted text).

REVISED ABSTRACT (216 words; max 250):

The transcription factor CREB is involved in a myriad of cellular functions in the central nervous system. For instance, the role of CREB via phosphorylation at the amino-acid residue Serine (Ser) 133 in expressing plasticity-related genes and activity-dependent neuronal plasticity processes has been extensively demonstrated. However, much less is known about the role of CREB phosphorylation at Ser 142 and 143. Here, we employed a viral vector containing a dominant negative form of CREB, with serine-to-alanine mutations at residue 142 and 143 to specifically block phosphorylation at both sites. We then transfected this vector into primary neurons in vitro or intra-cortically injected it into mice in vivo, to test if these phosphorylation events were important for activity-dependent plasticity. We demonstrated by immunohistochemistry of cortical neuronal cultures that the expression of Arc, a known plasticity-related gene, requires triple phosphorylation of CREB at Ser 133, 142, and 143. Moreover, we recorded visually-evoked field potentials in awake mice before and after a 7-day period of monocular deprivation to show that, in addition to CREB phosphorylation at Ser 133, ocular dominance plasticity in the visual cortex also requires CREB phosphorylation at Ser 142/143. Our findings suggest that Ser 142/143 phosphorylation is an additional post-translational modification of CREB that triggers the expression of specific target genes and activity-dependent neuronal plasticity processes.

2) There are two more supplementary figures explaining the virus constructs and validation. It would be helpful to add those figures to the main text to guide the reader through the experimental procedures and logical thinking about results. Supplementary Figure 1 could be Figure 2A, for example.

We agree with the reviewer that the schematics and validations will help the reader understand our experiments more clearly. We have moved all three supplementary figures into the main text.

3) Figure legends of supplementary data indicate three figures, but only two figures are found.

We thank the reviewer for noticing this, and we apologize for our oversight. We have included the third supplementary figure (now Figure 4 of the main text) in the revised version of the manuscript.

4) Section B in supplementary figure 2 is not explained in the figure legend.

Section A and B of Figure S2 were labeled as Left and Right in the figure legend. We have changed the legend labels to correctly reflect the figure labels in the revised manuscript. Note that the Figure S2 referred to here is now Figure 2B of the main text.

5) In the methods describing Western, how GFP of the virus reached the area of interest (that used to guide during dissection)? Was this under the control of a specific promoter? This should be clearly described in the virus description section.

The HSV constructs are not under a cell or region-specific promoter. For all in vivo experiments including the viral-mediated blockade, regional specificity was achieved via stereotaxic injection as described in the corresponding section of the methods. We have included the following sentence in the Western Blots section to direct the reader to the appropriate information - “The HSV construct was intracortically administered into Layer IV of the visual cortex of mice via stereotaxic injection (see Intracortical Injection section of the Methods below).”

6) Methods section lacks a clear description of cell counting/or OD such as; numbers/experiment for ICC analysis, criteria of choosing cells for analysis, etc. In this manuscript, just the number of cells is described in the figure legends (which is insufficient for such analysis and should be stated in the methods). The number of times the experiments were replicated should also be included- Figure legend should describe n of experiment/groups, not cells.

We agree the reviewer. We have added the following text to the Confocal imaging and analysis section of the methods to clarify how cells were manually selected and counted - “Using the Grid and Cell Counter tools in ImageJ software, all GFP-positive cells (GFP = virus expression) that co-expressed CamKII (Alexafluor 594; CamKII = marker of excitatory neurons) were manually selected and counted. Only virus-infected, excitatory neurons with clear somatic boundaries and a visible nucleus were included in this analysis, determined by colocalization of GFP and CaMKII.” We have also added the number of experiments per group to the figure legends.

Also, it is not indicated how the nucleus was defined to determine nuclear-increased phosphorylation of CREB (Figure 1). It would be advisable to show nuclear staining or tell how the nuclear area was defined.

We have added the following text to the Confocal imaging and analysis section of the methods to describe how the nuclear area was defined - “The localization of CamKII in the cytosol and dendrites allowed us to clearly delineate the nucleus of every cell analyzed. To determine nuclear staining, we used ImageJ to specifically define the nuclear region as the region of interest (ROI), and then quantified the CREB fluorescence within each ROI. The average optical density (OD) of the ROI was determined, and the background fluorescence was subtracted from the OD value of each cell.” Using the magnified insets of Figure 1 as a representative example - the dark circle of the nucleus seen in the blue channel (CamKII staining) was selected as the ROI for that cell, and the fluorescence within this ROI was quantified in the red channel as CREB staining. Group quantification and statistics confirmed what the images show - an increase in CREB nuclear staining in the +KCl condition.

7) In figure1, stimulation with potassium triggers Ca2+ influx and therefore activates CAMKII. This should be reflected as a change in CAMKII between stimulated vs control in immunocytochemistry (ICC) of figure 1.

The reviewer is correct in saying that KCl would trigger Ca2+ influx and activate CamKII. However, CamKII activation by the Ca2+/calmodulin complex can only be detected by its subsequent kinase activity, most commonly the autophosphorylation of its Thr286 residue (visualized using phospho-specific antibodies) (Pasek et al., 2015, Mol Cell Neurosci; Bossuyt and Bers, 2013, J Mol Med). Since we were only using CamKII as a marker to identify excitatory neurons in our cultures, we used a non-phosphorylated, “generic” CamKII antibody, which would detect all CamKII regardless of its activation status. Accordingly, we did not see a change in CamKII levels between stimulated and control groups of ICC.

8) Further analysis is needed, at least Western blot to confirm findings of figure 2 about ARC link to CREB. In addition, the early conclusions in the discussion, such as the following statement, need further confirmation. “We presented direct evidence that triple phosphorylation at Ser133, 142, and 143 is a mechanism for the activity-dependent induction of Arc by CREB”.

The requirement of CREB-Ser133 phosphorylation for Arc expression has been reported in the literature. One study showed that neurons infected with a dominant-negative viral vector containing the CREB-S133A mutation (prevents phosphorylation at Ser133) were significantly less likely to express any Arc protein compared to non-infected neighboring neurons. (Kim et al., 2013, Front Neural Circuits). More recently, another study showed that administration of KG-501 inhibitor (blocks the binding of Ser133 phosphorylated-CREB to its cofactor CBP), partially prevented the glutamate-induced elevation of Arc protein expression (Chen et al., 2017, Neurosci Lett).

However, there is also evidence that Arc mRNA levels did not increase in neurons infected with a constitutively-active-CREB viral vector (maintains S133 always phosphorylated) compared to non-infected neighboring neurons. This suggests that increasing Ser133 phosphorylation, in and of itself, is not sufficient to induce Arc expression. We proposed that CREB phosphorylation at Ser142/143 is an additional required event for Arc expression, and the ICC results shown in Figure 3 of the revised manuscript support this hypothesis.

Followed the reviewer’s suggestion we also have conducted another experiment (Fig 3C-D) showing that blockade of CREB phosphorylation at serine 142/143 reduced ARC expression in vivo (assessed by WB as suggested by the reviewer).

Minor concerns:

1. Why were cells infected at DIV 10 not at an earlier time point?

DIV 14 is a typical age at which neuronal plasticity experiments are conducted in culture, as this allows the cells sufficient time for synaptogenesis and to reach a relatively stable, mature state after initial plating. However, the number of cortical neurons in culture was reported to decrease significantly after DIV 14 (Harrill et al., 2015. Mol Brain). In order to find a balance between allowing sufficient synaptogenesis to study plasticity and maximizing the number of cells that would survive following virus infection, we started our manipulations after 10-11 days in culture. The virus was incubated with the cells overnight to allow adequate viral expression, and the KCl stimulation and subsequent fixation for ICC was done at DIV 12.

2. Typo on page 3 second paragraph “Taken together, these data show that Ser142/143 phosphorylation could lend specificity to CREB activation.”

We do not see any typos (spelling/grammar) in the sentence the reviewer has quoted here. However, to clarify the wording and better explain the idea that different CREB phosphorylation events could “lend specificity to CREB activation” by downstream expression of different gene pools, we have added/revised some text in the introduction. The new text is highlighted in bold.

3. Please revise citations and references; there are typos there too.

We have revised all the in-text citations and the reference list. Only newly added citations/references are in bold.

4. Please revise the work art of graphs, particularly fig.2, where the line is in the middle of the letter defining axis X, and Figure 3, where the n=of experiments float around and are not in the graph bars. It will be advisable that the format of all the graphs of the figures are the same.

We have revised the figures to fix all these errors.
